# Evaluation of Chemical Constituents and Important Mechanism of Pharmacological Biology in *Dendrobium* Plants

**DOI:** 10.1155/2015/841752

**Published:** 2015-04-06

**Authors:** Yau Lam, Tzi Bun Ng, Ren Ming Yao, Jun Shi, Kai Xu, Stephen Cho Wing Sze, Kalin Yanbo Zhang

**Affiliations:** ^1^School of Chinese Medicine, LKS Faculty of Medicine, The University of Hong Kong, 10 Sassoon Road, Hong Kong; ^2^School of Biomedical Sciences, Faculty of Medicine, The Chinese University of Hong Kong, Shatin, NT, Hong Kong

## Abstract

*Dendrobium* species, commonly known as “Shihu” or “Huangcao,” represents the second largest genus of Orchidaceae, which are used commonly as tonic herbs and healthy food in many Asian countries. The aim of this paper is to review the history, chemistry, and pharmacology of different *Dendrobium* species on the basis of the latest academic literatures found in Google Scholar, PubMed, Sciencedirect, Scopus, and SID.

## 1. Introduction

The origin of orchids (Orchidaceae) probably has to be backdated to 120 million years ago. It is the largest family group among angiosperms, as well as the most highly evolved family of the flowering plants, with approximately 25,000 to 35,000 species under 750 to 900 genera [1–4].* Dendrobium* species, commonly known as “Shihu” or “Huangcao,” is the second largest genus in Orchidaceae. Most* Dendrobium* species grow best in relatively high and mountainous areas, at 1400–1600 m above sea level, at a mild temperature, and in a humid and foggy environment. Characterized by a broad geographical distribution, which allows* Dendrobium* species to grow into tremendous diversities producing a large number of interspecific hybrids with different morphological features, they are widely distributed in Asia, Australia, and Europe, for instance, in India, Sri Lanka, China, Japan, Korea, New Guinea, and New Caledonia [5]. In the early age, using molecular approaches would delimit the subtribe-Dendrobiinae, affecting approximately 1100 species (900 in* Dendrobium* Sw.) from Indo-Asian and Pacific regions [6, 7], and more than 1200 species in Australasia are from various* Dendrobium* species. Today, Indo-Asian and Pacific regions have one of the largest and most diverse and taxonomically problematic orchid groups [8]. Interestingly, there has been a long history of the usage of first-rate herbs and folk traditional herbs in India and China [9].

Since the ancient times (600 B.C.) in India, the oldest references regarding the use of medicinal herbs are found in the Sanskrit literature, namely, “Charaka Samhita.” The earliest treatise on “Ayurveda” describes the property of plant drugs and their uses. In the Ayurvedic system of medicine,* Flickingeria macraei* is used in “Ayurveda.” It is commonly named as “jeevanti” and is used as an astringent to the bowels, as an aphrodisiac, and in asthma and bronchitis [10]. Other commonly used orchid drugs in the Ayurvedic system are salem, including jewanti (*Dendrobium alpestre*). Similarly, it is the first time for China to regard orchids as herbal medicines [11]. The Emperor “Shen-Nung” advised on the medicinal properties of* Dendrobium* species in “materia medica” in the 28th century B.C. [12]. As early as 200 B.C., the Chinese pharmacopoeia “The Sang Nung Pen Tsao Ching” mentioned* Dendrobium* as a source of tonic, astringent, analgesic, and anti-inflammatory substances [13]. In Song Dynasty (960–1279 A.D.). It mentioned the medicinal uses of orchids, namely* Dendrobium* species according A Diagnosis of Medical Herbs from the “Zheng Lei Ben Cao”. In Ming Dynasty (1368–1644) many references on the use of orchids as medicinal herbs were available [14].

## 2. The Chemical Compounds of* Dendrobium* Species

The stem of* Dendrobium* species has been used in traditional Chinese medicine as a tonic and antipyretic since ancient days for treating human disorders. However, misidentification and adulteration led to a loss of therapeutic potency and potential intoxication. For decades, fast developing molecular techniques using DNA fingerprinting, DNA sequencing, and DNA microarray have been applied extensively to authenticate Chinese medicinal materials, including various* Dendrobium* species [15], namely,* D. aphyllum*,* D. candidum*,* D. chrysanthum*,* D. densiflorum*,* D. huoshanense*,* D. gratiosissimum*,* D. longicornu*,* D. nobile*,* D. secundum*,* D. chrysotoxum*,* D. crystallinum*,* D. fimbriatum*, and others [16]. In the early years, Williams and Harborne conducted a major survey of leaf flavonoids at the Plant Science Laboratories of the University of Reading in UK. They conducted research on 142 species belonging to 75 genera and found that the most common constituents were flavone C-glycoside and flavonols [17]. Since then, about 100 compounds from 42* Dendrobium* species including 32 alkaloids, 6 coumarins, 15 bibenzyls, 4 fluorenones, 22 phenanthrenes, and 7 sesquiterpenoids constituents have been discussed and reviewed [18–31] (Table 1). To date, various* Dendrobium* species are known to produce a variety of secondary metabolites. The biological activities and pharmacological actions of all of these compounds were investigated in detail [32–39].

## 3. The Pharmacological Effects of* Dendrobium* Species

### 3.1. Antioxidant Activities

Oxidative stress is induced by free radicals that participate in a variety of chemical reactions with biomolecules, leading to a pathological condition. Reactive oxygen species (ROS) is one of the major free radicals. It mainly comprises superoxide (O_2_^−^) and nitric oxide (NO) radicals, including (1) catalase (CAT), (2) peroxidase (POD), (3) ascorbate peroxidase (APX), (4) 2,2′-azino-bis(3-ethylbenzothiazoline-6-sulphonic acid) or ABTS, (5) hydroxyl, and (6) 1,1-diphenyl-2-picrylhydrazyl (DPPH) radicals. During these processes, it was found that the content of malondialdehyde (MDA) was increased.

However, cells possess two distinctive antioxidant defense systems to counteract the damage, including enzymatic antioxidants and nonenzymatic antioxidants. Enzymatic antioxidants comprise catalase, superoxide dismutase (SOD), glutathione peroxidase (GPx), and others associated with enzymes/molecules. Nonenzymatic antioxidants include ascorbic acid (vitamin C), *α*-tocopherol (vitamin E), and *β*-carotene, which play a key role in removing reactive oxygen species.

Oxidative stresses have been classified as exogenous factors and endogenous factors. Factors related to environmental stress include water/soil drought stress, chilling injury stress, and sound wave stress. There were studies on the effects of cold storage on cut* Dendrobium* inflorescences, showing that chilling injury symptoms for floral buds and open flowers of* Dendrobium* could lead to oxidative stress, stemming from the production of reactive oxygen species. In this process, the decrease of cellular functions by peroxidation of membrane lipids when lipoxygenase enzymes are degraded in cells. However, this physiological process in floral buds and open flowers is part of antioxidant defense systems, which can decrease the content of oxygen free radicals and other oxygen compounds. It indicates the protective cellular functions and antioxidant capacity. This is an adaptive nature to ensure some plants survive in the freezing winter [62].

In addition, sound wave stress is one of the environmental stresses, similar to the low temperature stress. Studies of the effects of sound wave stress on* D. candidum* yielded data indicated it as a lipid peroxidation parameter which can regulate the levels of MDA. Firstly, the MDA content in different organs is increased and then declines afterwards, which will then be followed by an increase again. That gives a net increase of the level of MDA. It was noted that the MDA level appears to be the lowest when the activities of antioxidant enzymes are the highest. However, the levels of MDA are yet to be fully understood. We believe the antioxidant enzymes could protect plant cells from oxidative damage in sound wave stress [63]. However, the correlation between the mechanisms of antioxidant action and the sound wave stress and chilling injury stress has yet to be identified.

Water deficit caused by soil drought is one of the most frequent environmental stresses. The effects of exogenous drought menace, prompting further increases in the activity of ROS and decrease in the malondialdehyde (MDA) content, to prevent the breakage of DNA and protein degradation from doing damage to plant life [64]. It is well known that nitric oxide (NO) is a ubiquitous signal molecule involved in many life processes of plants, seed germination, hypocotyl elongation, leaf, expansion, root growth, lateral roots initiation, and apoptosis, and so forth. It is also involved in multiple plant responses to environmental stress. Data demonstrated that, at lower concentrations of exogenous NO with 50 mmol L^−1^ SNP, the activation of POD, SOD, and CAT was significantly increased, and the MDA content decreased with 50Ml SNP.NO could protect* D. huoshanense* against the oxidative insult caused by a drought stress; meanwhile, a high level of RWC can be maintained. Furthermore, it suggests that the molecular messenger NO can trigger epigenetic variation and increase the demethylation ratio of methylated sites for* D. huoshanense* by using DNA analysis. These results may imply that the expression of some genes is involved in the response to drought stress triggered by NO [65].

Numerous* Dendrobium* species such as* D. nobile*,* D. denneanum*,* D. huoshanense*,* D. chrysotoxum*,* D. moniliforme*,* D. tosaense*,* D. linawianum*,* D. candidum*,* D. loddigesii*, and* D. fimbriatum* have polysaccharides as the active compounds. Polysaccharides play important roles in many biological processes and are used to treat various diseases [66, 67]. Polysaccharides isolated from* D. nobile*,* D. huoshanense*,* D. chrysotoxum*, and* D. fimbriatum* species manifest antioxidant and free radical scavenging activities.* D. nobile* polysaccharide displayed the highest scavenging activity toward hydroxyl, ABTS, and DPPH free radicals [40, 68, 69]. However,* D. fimbriatum* polysaccharide has significant scavenging action toward ABTS free radicals, and it also shows a weak DPPH free radical scavenging action. On the contrary,* D. denneanum* polysaccharide exerts a powerful DPPH free radical scavenging action, but its ABTS scavenging effect is not obvious [54]. In addition, both* D. huoshanense* and* D. chrysotoxum* polysaccharides reveal an insufficient ABTS free radical scavenging effect. However, it manifests potential hydroxyl radical scavenging activity. Overall, the free radical scavenging activities of polysaccharides on hydroxyl and DPPH free radicals remain at a high level, but the inhibitory effect on ABTS free radicals is weak. The above results, compared with other types of polysaccharides from* D. denneanum* and* D. nobile*, are shown in Tables 2 and 3, and others are shown in Table 4 [41, 53]. The polysaccharide of* D. huoshanense* has a backbone of (1 → 4)-linked *α*-D-Glcp, (1 → 6)-linked *α*-D-Glcp, and (1 → 4)-linked *β*-D-Manp from mannose (Man), glucose (Glc), and a trace of galactose (Gal). Its antioxidant effect in the livers of CCl4-treated mice was evidenced by a decrease of malondialdehyde (MDA) and increase of superoxide dismutase (SOD), catalase (CAT), and glutathione peroxidase (GPx) [70].

#### 3.1.1. The Antioxidant Activities of Bibenzyl Derivatives, Phenanthrenes, and Stilbenes from* D. candidum* and* D. loddigesii*

Four new bibenzyl derivatives, dendrocandin F, dendrocandin G, dendrocandin H, and dendrocandin I, are extracted from* D. candidum*. They act as antioxidant agents to clear up the free radicals. Accordingly, the IC50 values of dendrocandin F and dendrocandin G are 55.8 mM and 32.4 mM, respectively [49]. A series of structurally related compounds from* D. loddigesii*, known as loddigesiinol A–D, suppress the production of nitric oxide (NO) with IC_50_ values of 2.6 mM for loddigesiinol A, 10.9 mM for loddigesiinol B, and 69.7 mM for loddigesiinol D. Further study indicated that phenanthrenes and dihydrophenanthrenesin* D. loddigesii* have significant effects on inhibiting the production of NO and DPPH free radicals. These compounds are known as (numbered as 1a–7a) phenanthrenes (1a), phenanthrenes (2a), phenanthrenes (3a), phenanthrenes (4a), dihydrophenanthrenes (5a), dihydrophenanthrenes (6a), and dihydrophenanthrenes (7a). Based on the above results, the scavenging activity of loddigesiinols toward NO free radicals is pronounced. The inhibitory actions of phenanthrenes and dihydrophenanthrenes from* D. loddigesii* on the production of NO and DPPH free radicals are recognised. The relevant data and information are shown in Table 5 [71].

### 3.2. Anti-Inflammatory Activity

#### 3.2.1. Inhibition of Nitric Oxide (NO) by* D. nobile* and* D. chrysanthum* Constituents

It is known that, upon lipopolysaccharide (LPS) stimulation, macrophages produce a large amount of inflammatory factors, such as tumor necrosis factor *α* (TNF-*α*), interleukin-1 beta (IL-1*β*), interleukin 6 (IL-6), interferon (IFN), and other cytokines. LPS induces endogenous nitric oxide (NO) biosynthesis through induction of inducible nitric oxide synthase (iNOS) in macrophages, which is involved in inflammatory responses [42].* D. nobile* and* D. chrysanthum* constituents strongly inhibit TNF-*α*, IL-1*β*, IL-6, and the NO production.

#### 3.2.2. *D. nobile* Constituents

From* D. nobile* two new bibenzyl derivatives, namely, nobilin D (given number 1) and nobilin E (given number 2), and a new fluorenone, namely, nobilone (given number 3), together with seven known compounds (given numbers 4–10), including R_1_ = R_2_ = OCH_3_ R_3_ = OH R_4_ = H (4), R_1_ = R_2_ = R_3_ = OCH_3_ R_4_ = H (5), R_1_ = R_2_ = OH R_3_ = R_4_ = H (6), R_1_ = R_3_ = OCH_3_ R_2_ = OH R_4_ = H (7), R_1_ = OCH_3_ R_2_ = R_3_ = OH R_4_ = H (8), R_1_ = R_3_ = OH R_2_ = R_4_ = H (9), and R_1_ = R_3_ = OH R_2_ = HR_4_ = OCH_3_ (10), have been identified. Compounds 1–3, 5, and 8–10 can inhibit NO production. On the other hand, compounds 2 and 10 can exhibit a strong to resveratrol, while enhanced cytotoxic potential has been found in compounds 4 and 7. Compounds 1–10 act as NO inhibitors, as evidenced by oxygen radical absorbency capacity (ORAC) assays. These findings are focused on all of these compounds, except compound 6, and their inhibitory effects on NO production, in murine macrophages (RAW 264.7), activated by LPS and interferon (IFN-ç) are shown in Table 5 [38]. Furthermore, it was found that a new phenanthrene, together with nine known phenanthrenes and three known bibenzyls, manifests an inhibitory effect on NO production in RAW 264.7 cells. The structures of all of the compounds (given numbers A–M), including R_1_ = R_3_ = OH, R_2_ = R_5_ = OCH_3_, R_4_ = H(A), R_1_ = R_4_ = R_5_ = OH, R_2_ = H, R_3_ = OCH_3_(B), R_1_ = R_5_ = OH, R_2_ = R_3_ = OCH_3_, R_4_ = H(C), R_1_ = R_2_ = OCH_3_, R_3_ = R_5_ = OH, R_4_ = H(D), R_1_ = H R_2_ = R_3_ = OH(E), R_1_ = H, R_2_ = OH, R_3_ = OCH_3_(F), R_1_ = OCH_3_, R_2_ = R_4_ = OH, R_3_ = R_5_ = H(G), R_1_ = R_3_ = OH, R_2_ = OCH_3_(H), R_1_ = R_5_ = OH, R_2_ = R_4_ = H, R_3_ = OCH_3_(I), R_1_ = R_3_ = OCH_3_, R_2_ = R_5_ = OH, R_4_ = H(J), R_1_ = R_2_ = R_4_ = OH, R_3_ = OCH_3_, R_5_ = H(K), R_1_ = OCH_3_, R_2_ = R_4_ = H, R_3_ = R_5_ = OH(L), and R_1_ = R_2_ = R_3_ = OH, R_4_ = R_5_ = H(M), were elucidated by analysis of the spectroscopic data, including extensive two-dimensional nuclear magnetic resonance spectroscopy (2D-NMR) and mass spectrometry (MS). All of compounds C, D, I, K, and L are relatively strong inhibitors of NO production, and their potencies are better than those of compounds A and E–H [58]. Compounds C, D, I, K, and L could inhibit NO synthesis which might contribute to the suppression of LPS-induced TNF-*α* and IL-1*β* production.

#### 3.2.3. *D. chrysanthum* Constituents

Anti-inflammatory compounds from ethyl acetate extracts of* D. chrysanthum* species were evaluated. These structures were elucidated on the basis of the high-resolution mass spectrometry, NMR spectroscopy, and X-ray crystal diffraction analysis. A novel phenanthrene phenol derivative with a spironolactone ring (dendrochrysanene), namely, 2-hydro-7,70,100-trihydroxy-4-40-dimethoxylspiro[(1H)-cyclopenta[a]naphthalene-3,30-(20H)-phenanthro[2,1-b]furan]-1,20-dione, was identified. Results showed anti-inflammatory activity of dendrochrysanene on iNOS mRNA induced by LPS in mouse peritoneal macrophages. Meanwhile, there are several inflammatory cytokines, such as TNF-*α*, IL-8, and IL-10, which were inhibited. Thus, the beneficial effects of dendrochrysanene compounds as anti-inflammatory agents were identified [72].

### 3.3. Sjögren's Syndrome (SS)

Sjögren's syndrome (SS) is a chronic autoimmune disease with disorder of the exocrine glands. The symptoms are dry eyes, dry mouth, dry throat and thirst with blurred vision, and so forth. The abnormal distribution of salivary glands in SS leads to an inflammatory effect and pathological processes triggering lymphocyte infiltration and apoptosis. Lymphocyte infiltration and apoptotic pathways, shown by regulation of Bax, Bcl-2, and caspase-3, have been reported in submandibular glands (SG). Furthermore, in pathological processes were also found that the subsequent signal of cell expression to mRNA of proinflammatory cytokines, such 30 as tumor necrosis (TNF-a), interleukin (IL-1*β*), and IL-6. As well as, a high level of expression of aquaporin-5 (AQP-5) is identified. AQP-5 is one of a water channel protein, and plays an important role in 31 the salivary secretions [73, 74].

**Figure 1:**
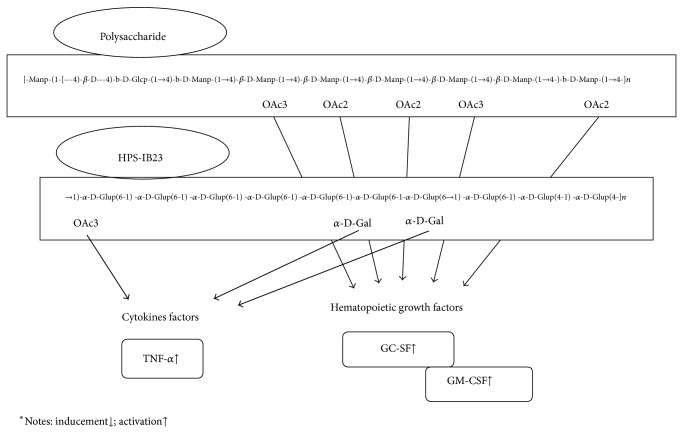
Cytokines activated by polysaccharide and polysaccharide (HPS-IB23) from* D. huoshanense*. The effect of two OAc3 and three Oac2 from polysaccharide on GC-SF and GM-CSF upregulation, and also the effect of one oAc3 and two *α*-D-Gal from polysaccharide (HPS-IB23) on TNF-*α* upregulation.

Treatment with* D. officinale* polysaccharides (DP) could suppress the progression of lymphocyte infiltration and apoptosis in SS and rectify the chaos of proinflammatory cytokines including TNF-*α*, IL-1 beta, and IL-6 in SG [71, 75] (Figure 1). mRNA expression of TNF-*α* was inhibited. The process of regulation resulted in a series of marked responses, such as translocation of NF-*κ*B, prolonged MAPK, cytochrome C release, and caspase-3 activation, which have been identified [55, 76]. In addition to the findings on DP treatment, there are reports on the findings on the extracts of* D. candidum* and* D. nobile*.

A clinical study found that, after 1 week of oral administration of the extract of* D. candidum*, salivary secretion was increased by approximately 65%. The extract promoted the expression of aquaporin-5 useful for treatment of Sjögren's syndrome (SS).

Chrysotoxine isolated from* D. nobile* can increase the expression of AQP-5 in dry eyes, one of the symptoms of SS, and restore the distribution of AQP-5 in lacrimal glands and corneal epithelia, by inhibiting the release of cytokines, such as IL-1, IL-6, and TNF-*α*. In the meantime, it can activate the mitogen-activated protein kinase (MAPK) signaling pathways. Furthermore, it elicits an increase in the production of matrix metalloproteinase-9 (MMP-9). Ultimately, it leads to an increase of saliva and tear secretion. The medical efficacy of the extracts of* D. officinale*,* D. nobile*, and* D. candidum *species has been demonstrated [43] (Figure 1).

### 3.4. Neuroprotective Effect (Parkinsonian Syndrome)

Five bioactive derivatives isolated from* Dendrobium* species, namely, chrysotoxine (CTX), moscatilin, crepidatin, nobilin B, and chrysotobibenzyl, are potential neuroprotective compounds with antioxidant activity which can be used in the treatment of Parkinson's disease (PD). The IC_50_ values of DPPH free radical scavenging activities of chrysotoxine (CTX), moscatilin, crepidatin, and nobilin B were 20.8 ± 0.9, 28.1 ± 7.1, 38.2 ± 3.5, and 22.2 ± 1.4, respectively. The above data show that crepidatin is better than other compounds in DPPH free radical scavenging capacity. CTX could selectively antagonize MPP^+^ in dopaminergic pathways in the brain; also 6-hydroxydopamine (6-OHDA) has been inhibited by mitochondrial protection and NF-*κ*B modulation in SH-SY5Y cells. Thus, it could explain how it may be beneficial in preventing PD [44, 77].

In addition, results of CTX compound in* Dendrobium nobile* Lindl. used in treatment of PD have also been clearly reported. We evaluated the pharmacokinetics of oral (100 mg/kg) and intravenous (25 mg/kg) administration of CTX preparation using high performance liquid chromatography-tandem mass spectrometric (HPLC-MS/MS) method in animal tests [78]. Results indicate efficacy and safety in treating PD conditions. The antioxidant mechanisms towards 6-OHDA and SH-SY5Y and regulation of antiapoptosis in cell signaling pathways were described [52].

### 3.5. Immunomodulatory Activity

Lymphocytes can be classified as T lymphocytes, B lymphocytes, and macrophages, respectively. They are important to immune cells and play a key role for reactions and responses in the immune system. Generally, there are various types of lymphocytes which are activated by very complex signal transduction pathways. Among all, the most common type is the action of lipopolysaccharides (LPS) in macrophages, which are able to produce many different kinds of proinflammatory cytokines, especially TNF-*α*, which is one of the main proinflammatory cytokines and plays a critical role in mediating signal transduction and stimulating the immune defense system [79]. The immunopotentiating action from* Dendrobium* species produced by concanavalin A- (Con A-) stimulated proliferation of splenocytes in mouse. Finding the part of, which the* D. officinalis* produced a more potent immunopotentiating action, although it did not provided related to information of biological activity [57].

Sesquiterpenes from* D. nobile* showed a comitogenic effect on Con A- and LPS-stimulated mouse splenocytes. Other chemical components, including polysaccharides and sesquiterpene glycosides were also confirmed. Related sesquiterpene glycosides with alloaromadendrane, emmotin, and picrotoxane type aglycones, namely dendroside A, dendronobilosides A.* In vitro* biological tests suggest the types of polysaccharides and sesquiterpene glycosides (given as dendrosides D–G) significantly promoted cell proliferation and more stimulation of mouse T and/or B lymphocytes [45, 80]. On the other hand, compounds from* D. moniliforme*, namely, dendroside A, dendroside C, and vanilloloside, were found to stimulate the proliferation of B cells and inhibit the proliferation of T cells* in vitro* [46] (Table 6).

In addition, the leaf, stem (cell walls), and mucilage isolated of* D. huoshanense* using chemical and enzymatic analyzed by chromatographic and spectroscopic methods, results demonstrated the bioactivity from polysaccharides and it's structure, HPS-1B23. The potential effects for main composed of more monosaccharides, showing Xyl, Ara, Man, Glc, Gal, and GalA, by HPS-1B23, signify an increasing immunostimulating activity through upregulating the levels of several cytokines, including TNF-*α*, IL-10, IL-6, and IL-1 [47]. In addition, the stem cell mucilage polysaccharides of* D. huoshanense* composed of *β*-(1-4)-D-Glcp and *β*-(1-4)-D-Manp linkages with partial acetylated mannosides were identified, which upregulated the growth factors GM-CSF and G-CSF. DHP-4A stimulated RAW 264.7 macrophages to secrete NO, TNF-*α*, IL-6, and IL-10 by activating p38, ERK, JNK, and NF-*κ*B. The results provide clear scientific evidence on regulation of hematopoietic growth factors and cytokines factors in the immune system by different polysaccharides from* D. huoshanense* [81, 82]. Interestingly, the total polysaccharides of* D. huoshanense* induced the expression of interleukin-1 receptor antagonist (IL-1ra) in human monocytes. Which the IL-1ra was regulated by 37 a series of signaling pathways, like ERK/ELK, p38 MAPK, PI3K, and NF-*κ*B [83].

In addition, the water-soluble polysaccharides (DTP) derived from* D. tosaense* were isolated by using HPAEC-PAD, HP-SEC, GC–MS, and NMR spectroscopy. They significantly increased the number of natural killer (NK) cells, NK cytotoxicity, macrophage phagocytosis, and cytokine induction in splenocytes, when administered orally to BALB/c mice for 3 weeks [59].

### 3.6. Treatment of Diabetes

The compounds isolated from* Dendrobium* huoshanense (DHP) have been evaluated for treatment of diabetes in recent years. The hypoglycemic and antioxidative activities of three polysaccharides, namely,* D. officinale* (DOP),* D. nobile* (DNP), and* D. chrysotoxum* (DCP), have been compared in diabetic mice. Result showed that the hypoglycemic effect of DHP was better than those of DNP and DOP. In addition, the antioxidant effect of DHP was better than those of DOP and DNP by regulating the superoxide dismutase, catalase, malonaldehyde, and l-glutathione levels in the liver and kidney. Thus, the hypoglycemic and antioxidant activities of DHP need to be studied more extensively in the future [60].

### 3.7. Antitumor Activity

#### 3.7.1. The Anticancer Effect of* D. chrysotoxum* and* D. nobile*

Fluorenone derivatives, namely, dendroflorin and denchrysan A, isolated from stems of* D. chrysotoxum* species, show significant inhibitory effects on the growth of human hepatoma BEL-7402 cells. The IC_50_ values of 1,4,5-trihydroxy-7-methoxy-9H-fluoren-9-one, denchrysan A 1, and dendroflorin were 1.49 *μ*g/mL, 1.38 *μ*g/mL, and 0.97 *μ*g/mL, respectively.

On the other hand, erianin, a phenanthrene from* D. chrysanthum*, is recognized as an antitumor agent. Reports showed that it has potent inhibitory activity in hepatoma Bel7402, melanoma A375, and HL-60 cells, as evidenced by inhibition of angiogenesis and induction of endothelial cytoskeletal disorganization* in vivo* and* in vitro*, but did not show antitumor activity [56, 61]. Thus, further investigation is needed to identify if the antitumor mechanisms of erianin and denbinobin are similar.

The antitumor effects of polysaccharides isolated from stems of* D. nobile*, namely, DNP-W, DNP-OH, and DNP-H, were studied. DNP-W was further fractionated to give six subfractions including DNP-W1, DNP-W2, DNP-W3, DNP-W4, DNP-W5, and DNP-W6 by using anion exchange chromatography. The results show that DNP-W, DNP-OH and DNP-H, DNP-W1, DNP-W2, and DNP-W3, especially DNP-W1 and DNP-W3, have a strong antitumor action in inhibiting sarcoma 180* in vivo* and HL-60 cells* in vitro* [48]. In addition, denbinobin is a major phenanthrene isolated from stems of* D. nobile*. The inhibitory mechanisms of denbinobin in SNU-484 cells have been observed. It significantly decreased the expressions of matrix metalloproteinase (MMP-2) and (MMP-9) and induced apoptosis through downregulation of Bcl-2 and upregulation of Bax in cancer cells. These findings may be used to provide reference for further studies on the pharmacological mechanisms from denbinobin and polysaccharides in* D. nobile* [50, 51, 84].

#### 3.7.2. Antilymphoma Activity of* Dendrobium formosum*

Lymphoma is a cancer of the immune system. The ethanolic extract of* Dendrobium formosum* showed antilymphoma activity* in vitro* as evaluated by the MTT assay. It also significantly prolonged survival time in Dalton's lymphoma bearing mice [85].

#### 3.7.3. Anti-Lung-Cancer Activity of* Dendrobium draconis*

The bibenzyl compound gigantol has been isolated from* Dendrobium draconis*. It inhibits several cancer cell lines and inhibits filopodia formation of the non-small-cell lung cancer cells. The molecular mechanism of gigantol includes (1) downregulating caveolin-1 (Cav-1), (2) activating ATP-dependent tyrosine kinase, and (3) regulating cell division cycle 42 (Cdc42) [86].

### 3.8. Antimicrobial Activity and Antifungal Activity

*Dendrobium* is an important economic plant; however, there have been certain cultivation issues yet to be resolved, particularly fungal infection [87].* Pythium vexans* has been reported to cause stem rot, crown rot, root rot, damping-off, and patch canker in* Dendrobium* plants [88]. The diseases could lead to damage to the seedlings of* D. chrysotoxum*,* D. chrysanthum*,* D. thyrsiflorum*, and* D. aurantiacum*, characterized by water-soaked, brown or yellowish lesions with a brown margin, resulting in dieback of the plants within a few days [89]. Interestingly, it also resulted in antimicrobial activity from endophytic bacteria isolated from* Dendrobium* species. Screening of various endophytic fungi from roots and stems of different* Dendrobium* species, including 53 endophytic fungi from* D. devonianum*; 23 endophytic fungi from* D. thyrsiflorum* by way of morphological and/or molecular biological methods, showed that 10 endophytic fungi from* D. devonianum* and 11 endophytic fungi from* D. thyrsiflorum* exhibited strong antimicrobial activity.* Phoma*,* Epicoccum*, and* Fusarium* isolated from two abovementioned* Dendrobium* species demonstrated antibacterial and antifungal activities. The pyrenophorol derivatives of* Phoma* possessed antagonistic activity against* E. coli*,* Bacillus megaterium*, and* Microbotryum violaceum*, and* Epicoccum* sp. slightly inhibited* S. aureus*,* E. coli*, and* B. subtilis*.* Fusarium* demonstrated inhibitory effect on different pathogens. Moreover, epicorazine A and epicorazine B derivatives of* Epicoccum nigrum* isolated from* D. thyrsiflorum* possessed antibacterial activity against* S. aureus* and* B. subtilis* and also inhibited the growth of Gram-positive and Gram-negative bacteria, with a potency stronger than that of ampicillin sodium [90–93].

### 3.9. Antimalarial and Antiherpetic Activities

#### 3.9.1. Antimutagenic Activity

Moscatilin obtained from* D. nobile* is a naturally occurring antimutagenic bibenzyl compound. This compound can exert a suppressive effect on the mutagenic activity of furylfuramide, 4-nitroquinoline-1-oxide (4NQO), N-methyl-N′-nitro-N-nitrosoguanidine, UV irradiation, 3-amino-1,4-dimethyl-5H-pyrido[4,3b]indole (Trp-P-1), benzo[a]pyrene (B[a]P), and aflatoxin B(1) [94].

### 3.10. Diabetic Retinopathy (DR)

Diabetes mellitus (DM) is one of the metabolic diseases. It can cause many complications, such as kidney failure, stroke, and foot ulcers. The ethanol extract of* Dendrobium chrysotoxum* (DC) used in treatment of diabetic retinopathy reduced the expression of a series of growing factors in DC-treated diabetic rats. It decreased the retinal mRNA expression level of vascular endothelial growth factor (VEGF), vascular endothelial growth factor receptor 2 (VEGFR2), serum growth factor (SVEGF), matrix metalloproteinase (MMP) 2/9, basic fibroblast growth factor (bFGF), platelet-derived growth factor (PDGF) A/B, insulin-like growth factor 1 (IGF-1), interleukin 1*β* (IL-1*β*), interleukin 6 (IL-6), and phosphorylation of p65. Moreover, a decrease in the expression of intercellular adhesion molecule-1 (ICAM-1) has been reported [95].

### 3.11. Antiplatelet Aggregating Activity

Studies on the aggregation of platelets induced through thrombin, arachidonic acid (AA), thrombin, collagen, and platelet-activating factor (PAF) were conducted by using 4 different aggregation inducers. Animal tests disclosed that different* Dendrobium* species including* Dendrobium loddigesii* and* Dendrobium densiflorum* possess antiplatelet aggregating activity. The results indicated some compounds isolated from two* Dendrobium* species more effectively inhibited AA-induced aggregation than they inhibited aggregation induced by PAF and collagen [96]. In animal tests, the antiplatelet aggregation of* D. loddigesii* species, which those compounds in part from moscatilin, moscatin and diacetate. They inhibit platelet aggregation was induced by AA was completely abolished by fraction 4 (50 *μ*g/mL) in rabbit platelets. Meanwhile, further demonstrated there are strongly inhibited both AA and collagen-induced platelet aggregations about 100 *μ*g/mL. However, in PAF there are no significant effects. In addition, comparison of different compounds from* D. densiflorum* species, such as moscatilin, homoeriodictyol, scoparone, scopoletin, and gigantol, which the antiplatelet aggregation activity of their were identified* in vitro*. Especially for scoparone has been reported to possess potent antiplatelet aggregation who more than other compounds. Thus, it possible interactied by compounds-compounds for antiplatelet aggregation [26, 97, 98] (Table 7).

## 4. Conclusion

In this paper, the history, chemistry, and pharmacology of different* Dendrobium* species are reviewed. Especially for biological pharmacology (Table 7). The pharmacological activities examined include antioxidant, anti-inflammatory, immunomodulatory, antitumor, antimicrobial/antifungal, antimutagenic activities, and antiplatelet aggregation activities. The ABTS, DPPH, and hydroxyl radical scavenging effects of different polysaccharides such as DNP, DNP2-1, DNP1-1, DNP3-1, and DNP4-2 have been investigated. Loddigesiinol, nobilone, dihydrophenanthrenes, and phenanthrenes have been evaluated for nitric oxide (NO) scavenging effect. Five types of polysaccharides from* D. huoshanense*,* D. chrysotoxum*, and* D. fimbriatum* species were compared.* D. huoshanense* and* D. chrysotoxum* polysaccharides exhibited stronger DPPH and hydroxyl scavenging activity while* D. fimbriatum* polysaccharides have stronger ABTS scavenging activity. Dihydrophenanthrenes and loddigesiinol D were isolated from* D. loddigesii* and* D. nobile* species. Dihydrophenanthrenes significantly inhibited DPPH production. Loddigesiinol D significantly inhibited NO production.

## Figures and Tables

**Table 1:** The compounds of various *Dendrobium* species.

Species	Constituent	Chemical formula	Structures of compound	Reference
*D. amoenum *				
	Bibenzyls derivatives			
	Amoenylin	R_1_ = R_3_ = Me, R_2_ = OH, R_4_ = H, R_5_ = OMe	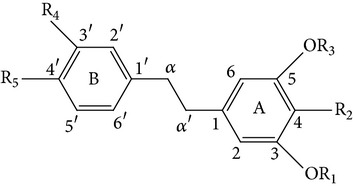	[19]
	Isoamoenylin	R_1_ = R_3_ = Me, R_2_ = OMe, R_4_ = OH, R_5_ = H		
	Moscatilin (analogues)	—		
	3,4′-Dihydroxy-5-methoxybibenzyl	—		
	—	R_1_ = R_2_ = R_4_ = H, R_3_ = Me, R_5_ = OH		
	—	R_1_ = R_3_ = Me, R_2_ = OH, R_4_ = H, R_5_ = OMe		
	—	R_1_ = R_3_ = Me, R_2_ = OAc, R_4_ = H, R_5_ = OMe		
	—	R_1_ = R_3_ = Me, R_2_ = OMe, R_4_ = OAc, R_5_ = H		
	—	R_1_ = Ac, R_2_ = R_4_ = H, R_3_ = Me, R_5_ = OAc		
	—	R_1_ = R_3_ = Me, R_2_ = R_5_ = OH, R_4_ = OMe		
	—	R_1_ = R_3_ = Me, R_2_ = R_4_ = OMe, R_5_ = OH		
	—	R_1_ = R_3_ = Me, R_2_ = R_5_ = OMe, R_4_ = OAc		
	—	R_1_ = R_2_ = R_5_ = H, R_3_ = Me, R_4_ = OH		
	—	R_1_ = Ac, R_2_ = R_5_ = H, R_3_ = Me, R_4_ = OAc		
	—	R_1_ = R_2_ = R_3_ = R_4_ = H, R_5_ = OH		

*D. candidum *				
	Bibenzyls derivatives			
	Dendrocandin C ((*S*)-3,4,4-trihydroxy-5,*α*-dimethoxybibenzyl) Dendrocandin D ((*S*)-3,4,4-trihydroxy-5-methoxy-*α*-ethoxybibenzyl) Dendrocandin E (3,3,4,4-tetrahydroxy-5-methoxybibenzyl)	R_1_ = R_2_ = R_5_ = OH, R_3_ = R_6_ = OCH_3_, R_4_ = H R_1_ = R_2_ = R_5_ = OH, R_3_ = OCH_3_, R_6_ = OCH_2_, CH_3_, R_4_ = H R_1_ = R_2_ = R_4_ = R_5_ = OH, R_3_ = OCH_3_, R_6_ = H	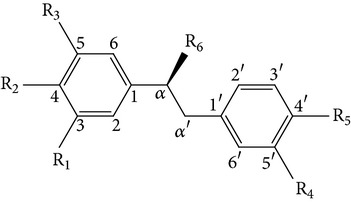	[20]
	Amotin (type 1, sesquiterpenoids)	—	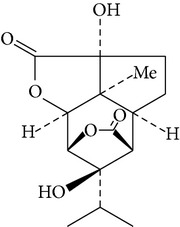	[19]
	Amoenin (type 2 sesquiterpenoids)	—	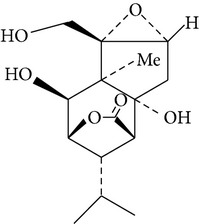	[19]
	Flaccidin (9,10-dihydrophenanthropyran)	—	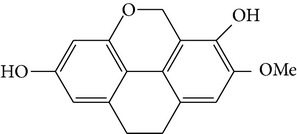	[19]
	Bibenzyls derivatives			
	4,4-Dihydroxy-3,5-dimethoxybibenzyl 3,4-Dihydroxy-5,4 dimethoxybibenzyl 3-*O*-Methylgigantol Dendrophenol Gigantol New compounds Dendrocandin A ((*R*)-3,4-dihydroxy-5,4,*α*-trimethoxybibenzyl)	R_2_ = R_5_ = OH, R_1_ = R_3_ = OCH_3_, R_4_ = R_6_ = OH R_1_ = R_2_ = OH, R_3_ = R_5_ = OCH_3_, R_4_ = R_6_ = OH R_1_ = OH, R_3_ = R_4_ = R_5_ = OCH_3_, R_2_ = R_6_ = H R_2_ = R_4_ = R_5_ = OH, R_1_ = R_3_ = OCH_3_, R_6_ = H R_1_ = R_5_ = OH, R_3_ = R_4_ = OCH_3_, R_2_ = R_6_ = H R_1_ = R_2_ = OH, R_3_ = R_5_ = R_6_ = OCH_3_, R_4_ = H	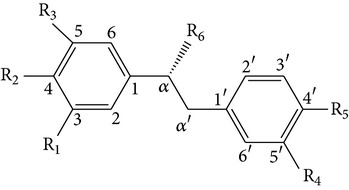	[21]
	Dendrocandin B (4-[2-[(2*S*,3*S*)-3-(4-hydroxy-3,5-dimethoxyphenyl)-2-hydroxymethyl-8-methoxy-2,3-dihydrobenzo[1,4]dioxin-6-yl]ethyl]-1-methoxyl benzene)	—	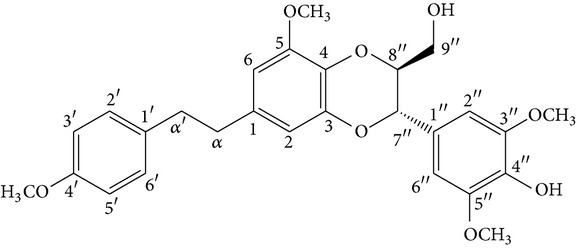	[21]

*D. aphyllum *				
	Aromatic compounds			
	Flavanthrin	—	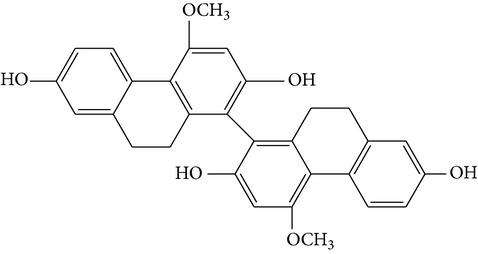	[22]
	Coelonin Iusianthridin	R_1_ = OH, R_2_ = OMe R_1_ = OMe, R_2_ = OH	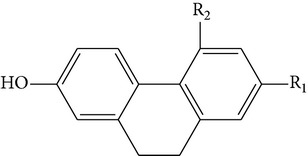	[22]
	Moscatin	—	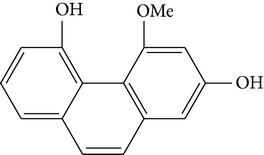	[22]
	Gigantol Batatasin III	R_1_ = R_3_ = OMe, R_2_ = OH R_1_ = OH, R_2_ = H, R_3_ = OMe	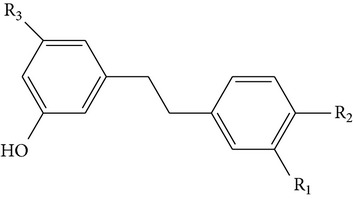	[22]
	Dibutyl phthalate Diisobutyl phthalate	R = CH_2_CH_2_CH_2_CH_3_ R = CH_2_CH(CH_3_)_2_	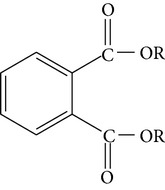	[22]
	*P*-hydroxyphenylpropionic methyl ester	—	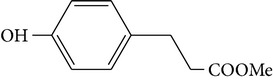	[22]

*D. cariniferum *				
	Phenanthrenequinone			
	Dendronone (5-hydroxy-7-methoxy-9,10-dihydro-1,4-phenanthrenequinone)	—	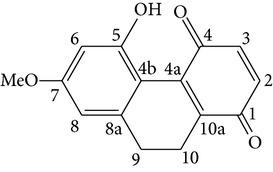	[23]

*D. chrysotoxum *				
	Aromatic organic compounds			
	(Fluorenones)			
	Dendroflorin Denchrysan A	R_1_ = H, R_2_ = OH R_1_ = OH, R_2_ = H	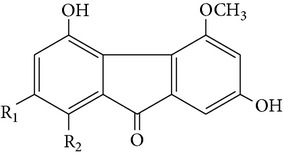	[24]
	1,4,5-trihydroxy-7-methoxy-9H-fluoren-9-one	—	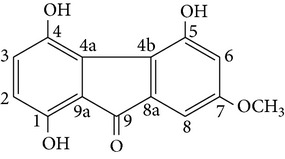	[24]
	(Fluorenol)			
	(9R)-4methoxy-9H-fluorene-2,5,9-triol	R = H	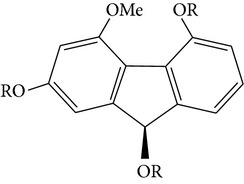	[25]
	Phenanthrene			
	2,7-dihydroxy-8methoxyphenanthro[4,5-bcd]pyran-5(5H)-one	—	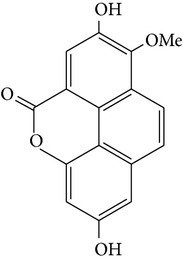	[25]

*D. densiflorum *				
	Bibenzyl			
	Densiflorol A	—	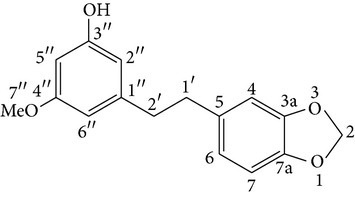	[26]
	Phenanthrenedione			
	Densiflorol B	R = H	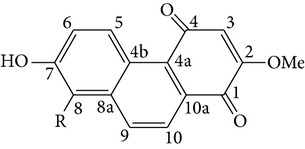	[26]
	Dendroflorin	R = OH	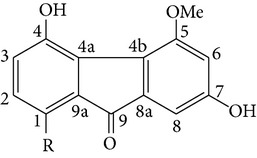	[26]
	Known as oleanolic acid and b-sitosterol compounds			
	(A) Dengibsin	R = H	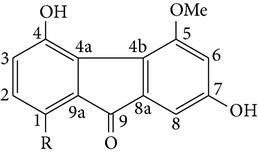	[26]
	(B) Cypripedin	R = OMe	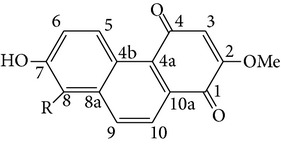	[26]
	(C) Gigantol (D) Moscatilin (E) Tristin	R = OH, R = H, R = OMe R = R = OMe, R = OH R = R = OH, R = H	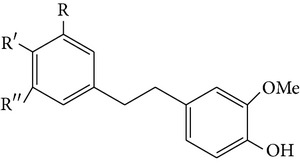	[26]
	(F) Naringenin (G) Homoeriodictyol	R = H R = OMe	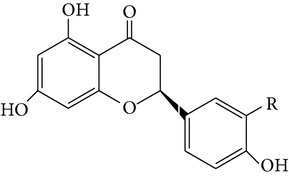	[26]
	(H) Moscatin	—	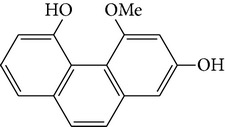	[26]
	(I) 2,6-dihydroxy-1,5,7-trimethoxyphenanthrene	—	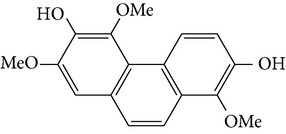	[26]
	(J) 4,7-dihydroxy-2-methoxy-9,10-dihydrophenanthrene	—	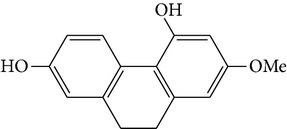	[26]
	(K) Scoparone (L) Scopoletin (M) Ayapin	R = R = OMe R = OMe, R = OH R + R = OCH_2_O	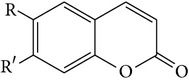	[26]

*D. longicornu *				
	Monoaromatic			
	compounds			
	Bis(2-ethylhexyl)phthalate Dibutyl phthalate	CH_2_CH(C_2_H_5_)(CH_2_)_3_CH_3_ (CH_2_)_3_CH_3_	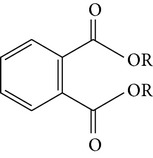	[27]
	Ethyl haematommate	C_11_H_12_O_5_	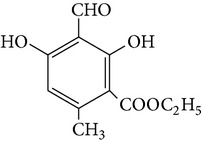	[27]
	Methyl *B*-orcinol carboxylate	C_10_H_12_O_4_	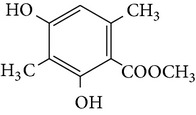	[27]
	*N*-Docosyl *trans*-ferulate Ferulaldehyde	R = COOCH_2_(CH_2_)_20_CH_3_ R = CHO	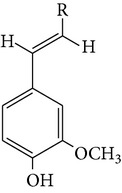	[27]

*D. nobile *				
	Sesquiterpenes			
	(Dendronobilins A)			
	Copacamphane-type	(1R,2R,4S,5S,6S,8S,9R)-2,8-Dihydroxycopacamphan-15-one	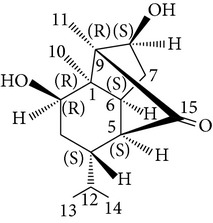	[28]
	Picrotoxane-type	(2b,3b,4b,5b)-2,4,11-Trihydroxy-picrotoxano-3(15)-lactone	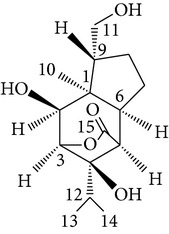	[28]
	Picrotoxane-type	(2b,3b,5b,9a,11b)-2,11-Epoxy-9,11,13-trihydroxypicrotoxano3(15)-lactone	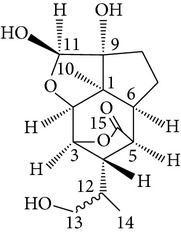	[28]
	Picrotoxane-type	(2b,3b,5b,12R^*^)-2,11,13-Trihydroxy-picrotoxano-3(15)-lactone	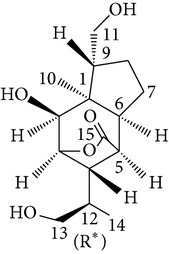	[28]
	Picrotoxane-type	(2b,3b,5b,12S^*^)2,11,13-Trihydroxy-picrotoxano-3(15)-lactone	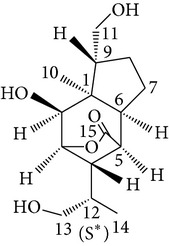	[28]
	Picrotoxane-type	(2b,3b,5b,9a)-,10-Cyclo-2,11,13-trihydroxy-picrotoxano-3(15)-lactone	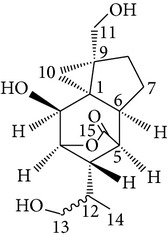	[28]
	Muurolene-type	(9b,10a)-Muurol-4-ene-9,10,11-triol	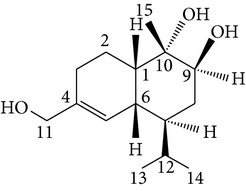	[28]
	Alloaromadendrene-type	(10a)-Alloaromadendrene-10,12,14-triol	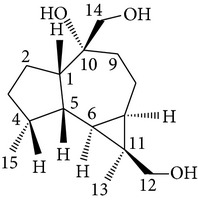	[28]
	Cyclocopacamphane-type	(5b)-Cyclocopacamphane-5,12,15-triol	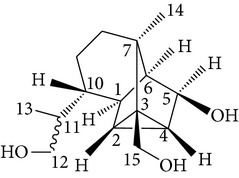	[28]
	Bibenzyl derivatives			
	Dendronophenol A	2,2′,9,9′-Tetramethoxy13′,14′-peroxy-1,1′-bis(bibenzyl)-6,6′-diol)	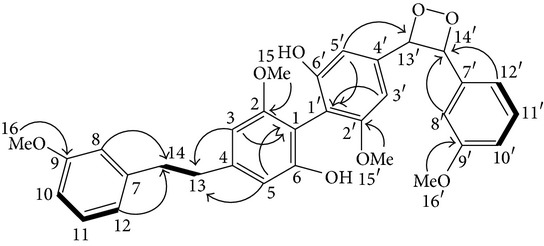	[29]
	Dendronophenol B	1-(2′,6′-Dimethoxy7′,8′-peroxyphenyl-propyl)-2,9-dimethoxybibenzyl-6,9′-diol)	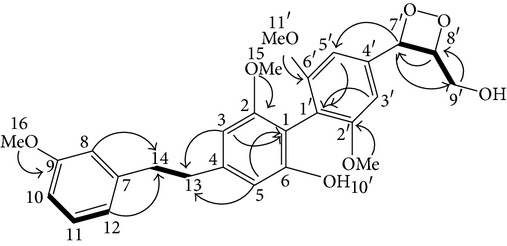	[29]

*D. thyrsiflorum *				
	Bi-bicyclic and bi-tricyclic compounds			
	Denthyrsin	[3-(5′,6′-Dimethoxybenzofuran-2′-yl)-6,7-dimethoxy-2H-chromen-2-one	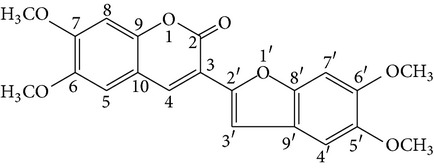	[30]
	Denthyrsinol	(4,5′-Dimethoxy-[1,1′]biphenanthrenyl-2,5,4′,7-tetraol	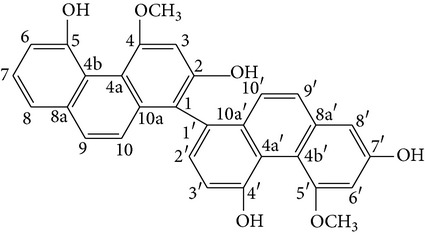	[30]
	Denthyrsinone	(7,4′,7′-Trihydroxy-2,2′,8′-trimethoxy-[5,1′]biphenanthrenyl-1,4-dione)	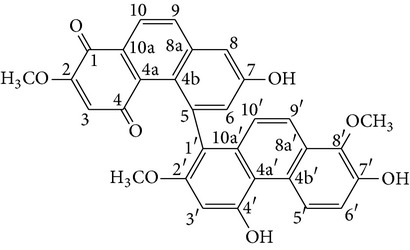	[30]
	Denthyrsinin	1,5,7-Trimethoxyphenanthrene-2,6-diol	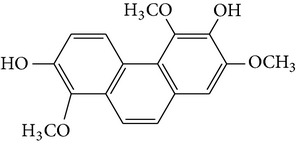	[30]
	Scoparone	—	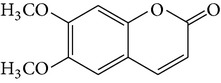	[30]
	b-Sitosterol	—	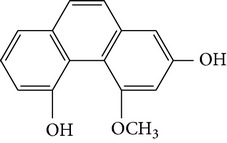	[30]
	Daucosterol	—	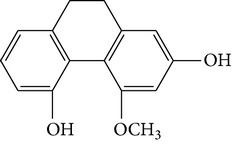	[30]

**Table 2:** Polysaccharides DDP1-1, DDP2-1, and DDP3-1 isolated from *D.  denneanum* and DNP1-1 DNP2-1, DNP3-1, and DNP4-2 isolated from *D.  nobile*.

	ABTS radicals	Hydroxyl radicals	DPPH radicals
*D. denneanum *			
DDP1-1	(LW)	(LW)	(LW)
DDP2-1	(LW)	(HH)	(HH)
DDP3-1	(LW)	(LW)	(LW)
*D. nobile *			
DNP1-1	(LW)	(LW)	(LW)
DNP2-1	(LW)	(LW)	(LW)
DNP3-1	(LW)	(LW)	(LW)
DNP4-2	(HH)	(HH)	(HH)

^∗^Notes: Low (LW); High (HH).

Up the table: the antioxidant effect of DNP4-2 on ABTS, hydroxyl, and DPPH free radicals, suggesting the levels of DNP4-2 higher than DNP1-1, DNP2-1, and DNP3-1; however, the antioxidant effects of DDP2-1 on hydroxyl and DPPH free radicals were higher than ABTS free radicals. Moreover, the DDP1-1 and DDP 3-1 on inhibitory effect were low than other compounds. We believe that the focus on antioxidant potential of DNP4-2 and DDP2-1 structures in near future.

**Table 3:** Assessment of the ABTS, hydroxyl, and DPPH scavenging abilities of DNP and compounds DNP4-2, DNP2-1, DNP3-1, and DNP1-1 derived from DNP.

DNP, DNP4-2, DNP2-1, DNP3-1, and DNP1-1				

0.5 mg for DPPH scavenging (%)			1 mg for DPPH scavenging (%)	
Type	Scavenging (%)		Type	Scavenging (%)

DNP	20		DNP	20–30
DNP4-2	20–30		DNP4-2	30–40
DNP2-1	5–10		DNP2-1	10–20
DNP3-1	About 5		DNP3-1	<5
DNP1-1	<5		DNP1-1	About 5

0.5 mg for ABTS scavenging (%)			1 mg for ABTS scavenging (%)	
Type	Scavenging (%)		Type	Scavenging (%)

DNP	30–40		DNP	<60
DNP4-2	About 40		DNP4-2	>60
DNP2-1	<20		DNP2-1	About 40
DNP3-1	≥20		DNP3-1	30–40
DNP1-1	10		DNP1-1	30

0.5 mg for hydroxyl scavenging (%)			1 mg for hydroxyl scavenging (%)	
Type	Scavenging (%)		Type	Scavenging (%)

DNP	30–40		DNP	35–40
DNP4-2	20–30		DNP4-2	30–35
DNP2-1	10		DNP2-1	10–15
DNP3-1	5–10		DNP3-1	10
DNP1-1	0		DNP1-1	5

Based on data for DNP, DNP4-2, DNP2-1, DNP3-1, and DNP1-1 scavenging (%)				

DPPH scavenging % (0.5 mg content): DNP4-2 > DNP > DNP2-1 > DNP3-1 > DNP1-1				
DPPH scavenging % (1 mg content): DNP4-2 > DNP > DNP2-1 > DNP1-1 > DNP3-1				

ABTS scavenging % (0.5 mg content): DNP4-2 > DNP > DNP3-1 > DNP2-1 > DNP1-1				
ABTS scavenging % (1 mg content): DNP4-2 > DNP > DNP2-1 > DNP3-1 > DNP1-1				

Hydroyl scavenging % (0.5 mg content): DNP > DNP4-2 > DNP2-1 > DNP3-1 > DNP1-1				
Hydroyl scavenging % (1 mg content): DNP > DNP4-2 > DNP2-1 > DNP3-1 > DNP1-1				

Up the table: it is shown that DPPH and ABTS free radical scavenging (%) of DNP4-2 are better than other compounds; however, the hydroxyl free radical scavenging (%) of DNP is better than other compounds.

**Table 4:** Comparison of the DPPH, hydroxyl, and ABTS free radical scavenging abilities of polysaccharides from *D.  huoshanense*, *D.  chrysotoxum*, and *D.  fimbriatum* species.

*Dendrobium* species	Chemical compounds	DPPH concentration (mg/mL)			
		0.5%	1%	2%	3%

*D. huoshanense* and *D. chrysotoxum *	Polysaccharides (scavenging effects %)	45–50	AB 80	90–95	—
*D. fimbriatum *	Polysaccharides (scavenging effects %)	—	10	—	40–50

*Dendrobium* species	Chemical compounds	Hydroxyl concentration (mg/mL)			
		0.5%	1%	2%	3%

*D. huoshanense* and *D. chrysotoxum *	Polysaccharides (scavenging effects %)	50	60–65	80	—
*D. fimbriatum *	Polysaccharides (scavenging effects %)	—	55–60	—	70–75

*Dendrobium* species	Chemical compounds	ABTS concentration (mg/mL)			
		0.5%	1%	2%	3%

*D. huoshanense* and *D. chrysotoxum *	Polysaccharides (scavenging effects %)	AB 10	AB 20	AB 25	—
*D. fimbriatum *	Polysaccharides (scavenging effects %)	—	AB 70	—	AB 90

^∗^Notes: —: no tests and about: AB.

The scavenging hydroxyl free radicals abilities of polysaccharides from *D.  huoshanense* and *D.  chrysotoxum* are stronger than *D.  fimbriatum*, and the scavenging ABTS free radicals abilities of polysaccharides from *D.  fimbriatum* are stronger than *D.  huoshanense* and *D.  chrysotoxum* polysaccharides. Results showed that polysaccharides (scavenging DPPH effects %) from *D.  huoshanense* and *D.  chrysotoxum* are higher than hydroxyl and ABTS. And polysaccharides (scavenging ABTS effects %) from *D.  fimbriatum* are higher than DPPH and hydroxyl free radicals.

**Table 5:** Inhibition of compounds from *D.  loddigesii* and *D.  nobile* species on NO and DPPH formation.

Species	Chemical constituent	NO	DPPH
*D. loddigesii*	Phenanthrenes (1a)	2.6	26.1
	Phenanthrenes (2a)	6.4	59.8
	Phenanthrenes (3a)	5.3	12
	Phenanthrenes (4a)	10.9	—
	Dihydrophenanthrenes (5a)	4.6	62.2
	Dihydrophenanthrenes (6a)	29.1	—
	Dihydrophenanthrenes (7a)	29.2	—
	Loddigesiinols A	2.6	—
	Loddigesiinols B	10.9	—
	Loddigesiinols C	—	23.7
	Loddigesiinols D	69.7	—

*D. nobile*	Nobilin D (1)	15.3	—
	Nobilin E (2)	19.2	—
	Nobilone F (3)	38.1	—
	Phenanthrenes and bibenzyls related-composition* *(4–10)		
	ROR_1_ = R_2_ = OCH_3_ R_3_ = OH R_4_ = H (4)	—	—
	ROR_1_ = R_2_ = R_3_ = OCH_3_ R_4_ = H (5)	48.2	—
	ROR_1_ = R_2_ = OH R_3_ = R_4_ = H (6)	—	—
	ROR_1_ = R_3_ = OCH_3_ R_2_ = OH R_4_ = H (7)	—	—
	ROR_1_ = OCH_3_ R_2_ = R_3_ = OH R_4_ = H (8)	36.8	—
	ROR_1_ = R_3_ = OH R_2_ = R_4_ = H (9)	32.9	—
	ROR_1_ = R_3_ = OH R_2_ = H R_4_ = OCH_3_ (10)	13.4	—

^∗^Notes: —: no tests; RO: relation composition.

Inhibitory effects of different compounds from *D.  loddigesii* and *D.  nobile* species on NO production. Result shown in Table 5, ranked from high to low as follows: loddigesiinol D > R_1_ = R_2_ = R_3_ = OCH_3_ R_4_ = H (5) > nobilone F (3) > R_1_ = OCH_3_ R_2_ = R_3_ = OH R_4_ = H (8) > R_1_ = R_3_ = OH R_2_ = R_4_ = H (9) > dihydrophenanthrenes (7a) > dihydrophenanthrenes (6a) > nobilin E (2) > nobilin D (1) > R_1_ = R_3_ = OH R_2_ = H R_4_ = OCH_3_ (10) > phenanthrenes (4a) = loddigesiinols B > phenanthrenes (2a) > phenanthrenes (3a) > dihydrophenanthrenes (5a) > phenanthrenes (1a) > loddigesiinols A, and all of the compounds inhibit DPPH prodruction (from high to low): dihydrophenanthrenes (5a) > phenanthrenes (2a) > phenanthrenes (1a) > loddigesiinol C > phenanthrenes (3a).

**Table 6:** The chemical compounds from *D.  nobile* and *D.  moniliforme* that inhibited/activated T cells and B cells.

*Dendrobium* species	Compound/structure	Lymphocytes	
		T cell	B cell
*D. nobile *	Dendroside D	(AD)	(AD)
	Dendroside E	(AD)	(AD)
	Dendroside F	(AD)	(AD)
	Dendroside G	(AD)	(AD)

*D. moniliforme *	Dendroside A	(IY)	(AD)
	Dendroside C	(IY)	(AD)
	Vanilloloside	(IY)	(AD)
	Denbinobin	(IY)	(AD)
	2,6-Dimethoxy 1,4,5,8-phenanthradiquinone	(IY)	(AD)

^∗^Notes: activation: AD; inhibition: IY.

Up the table, showed T cell and B cell activated by chemical compounds from *D.  nobile*, and inhibit T cell and activated on B cell by chemical compounds from *D.  moniliforme*.

**Table 7:** The pharmacological effects of *Dendrobium* species.

Species	Chemical compounds/structures	Pharmacological activities	References
*D. nobile *	Polysaccharides	Scavenging effect of hydroxyl, ABTS, and DPPH	[40, 41]
(SR)	Nobilin D, nobilin E, nobilone	Inhibitory on NO production	[42]
(SR)	Phenanthrenes	Inhibition of LPS-induced of nitric oxide production	[38]
(SR)	Dendroside A, Dendronobilosides A	Immunomodulatory activity	[43, 44]
(SR)	Glycosides (dendrosides D–G)	Significant Stimulation of the proliferation of mouse T and/or B lymphocytes	[43, 44]
(SR)	Polysaccharides (DNP-W1, DNP-W2, DNP-W3, DNP-W4)	Anticancer activity	[45]
(SR)	4,7-Dihydroxy-2-methoxy-9,10-dihy	Anticancer activity:	[46]
	Drophenanthrene, denbinobin	(SNU-484 human gastric cancer cells)	[46]
(SR)	(SR)	(human lung carcinoma), SK-OV-3	[47]
(SR)	(SR)	(human ovary adenocarcinoma)	[47]
(SR)	(SR)	HL-60 (human promyelocytic leukemia) cell lines	[47]
(SR)	Bibenzyl	Inhibit on furylfuramide	[48]
(SR)	(SR)	4-nitroquinoline-1-oxide (4NQO)	[48]
(SR)	(SR)	N-Methyl-N′-nitro-N-nitrosoguanidine	[48]
(SR)	(SR)	UV irradiation	[48]
(SR)	(SR)	3-Amino-1,4-dimethyl-5H-pyrido[4,3b]indole (Trp-P-1)	[48]
(SR)	(SR)	Benzo[a]pyrene (B[a]P)	[48]
(SR)	(SR)	Aflatoxin B(1)	[48]
(SR)	Nobilin D	Anti-inflammatory activity	[42]
(SR)	Nobilin E	(SR)	[42]
(SR)	Nobilone	(SR)	[42]
(SR)	R_1_ = R_2_ = OCH_3_ R_3_ = OH R_4_ = H	(SR)	[42]
(SR)	R_1_ = R_2_ = R_3_ = OCH_3_ R_4_ = H	(SR)	[42]
(SR)	R_1_ = R_3_ = OCH_3_ R_2_ = OH R_4_ = H	(SR)	[42]
(SR)	R_1_ = OCH_3_ R_2_ = R_3_ = OH R_4_ = H	(SR)	[42]
(SR)	R_1_ = R_3_ = OH R_2_ = R_4_ = H	(SR)	[42]
(SR)	R_1_ = R_3_ = OH R_2_ = H R_4_ = OCH_3_	(SR)	[42]
*D. loddigesii *	Phenanthrenes	Inhibition of nitric oxide (NO)	[49]
(SR)	Loddigesiinols A–D	(SR)	[49]
(SR)	Moscatilin, moscatin, moscatilin diacetate	Inhibited both AA and collagen-induced platelet aggregations	[50, 51]
(SR)	—	Inhibition of aggregation of rabbit platelets induced by arachidonic acid and collagen	[50, 51]
*D. huoshanense *	Polysaccharides	Inducing several cytokines, including IFN-c, IL-10, IL-6, and IL-1	[52]
(SR)	(SR)	Immunostimulating activity	[52]
(SR)	(SR)	Increase of TNF-*α* production	[52]
(SR)	(SR)	Inducing several cytokines, including hematopoietic growth factors GM-CSF and GCSF	[52]
*D. denneanum *	Polysaccharides	Scavenging effect of hydroxyl, ABTS, and DPPH	[53]
*D. fimbriatum *	Polysaccharides	Scavenging effect of hydroxyl, ABTS, DPPH	[54]
*D. candidum *	Bibenzyl	Antioxidant activity	[20]
*D. officinalis *	—	Immunomodulatory activity	[55]
*D. findlayanum *	—	Inhibition of *Alternaria alternata* and other	[56]
*D. densiflorum *	Moscatilin, homoeriodictyol, scoparone, scopoletin, gigantol	Antiplatelet aggregation activity	[50, 51]
*D. nobile*, *D. chrysanthum *	Erianin	Anticancer activity: hepatoma Bel7402 melanoma A375 and HL-60 cells	[57]
*D. chrysanthum*, *D. huoshanense *	Dendrochrysanene	TNF-*α*, IL-8, IL-10, and iNOS mRNAs were induced readily from mouse peritoneal macrophages by LPS	[58]
*D. devonianum*, *D. thyrsiflorum *	*Epicoccum* sp., epicorazine A Epicorazine B	Inhibition of *S. aureus*, *E. coli*, and *B. subtilis *	[59, 60]
*D. devonianum*, *D. thyrsiflorum *	Pyrenophorol derivatives	Inhibition of *E.coli*, *Bacillus megaterium*, and *Microbotryum violaceum *	[61]

^∗^Notes: —: no tests; SR: similar.
